# Co-construction of the family-focused support conversation: a participatory learning and action research study to implement support for family members whose relatives are being discharged for end-of-life care at home or in a nursing home

**DOI:** 10.1186/s12904-020-00647-5

**Published:** 2020-09-21

**Authors:** Sue Duke, Natasha Campling, Carl R. May, Susi Lund, Neil Lunt, Hospital to Home Co-researcher group, Gemma Bartlett, Lucy Harris, Elizabeth Flannery, Michael Connolly, Pam Booth, Gillian Galpin, Emma Wells, Elizabeth Price, Alison Faulkner-Butcher, Leanne Petch, Chris Ward, Alison Richardson

**Affiliations:** 1 School of Health Sciences, University of Southampton, Highfield, Southampton, SO17 1BJ England; 2 Faculty of Public Health and Policy, London School of Hygiene and Tropical Medicine, 15-17 Tavistock Place, London, WC1H 9SH England; 3 Department of Social Policy and Social Work, University of York, Heslington, York, YO10 5DD England; 4 University Hospitals Southampton and School of Health Sciences, University of Southampton, Highfield, Southampton, SO17 1BJ England

**Keywords:** End-of-life care, Family support, Family-Focused Support Conversation, Family Sense of Coherence, Acute hospitals, Normalization Process Theory, Participatory Learning and Action Research, Implementation

## Abstract

**Background:**

Many people move in and out of hospital in the last few weeks of life. These care transitions can be distressing for family members because they signify the deterioration and impending death of their ill relative and forthcoming family bereavement. Whilst there is evidence about psychosocial support for family members providing end-of-life care at home, there is limited evidence about how this can be provided in acute hospitals during care transitions. Consequently, family members report a lack of support from hospital-based healthcare professionals.

**Methods:**

The aim of the study was to implement research evidence for family support at the end-of-life in acute hospital care. Informed by Participatory Learning and Action Research and Normalization Process Theory (NPT) we co-designed a context-specific intervention, the Family-Focused Support Conversation, from a detailed review of research evidence. We undertook a pilot implementation in three acute hospital Trusts in England to assess the potential for the intervention to be used in clinical practice. Pilot implementation was undertaken during a three-month period by seven clinical co-researchers - nurses and occupational therapists in hospital specialist palliative care services. Implementation was evaluated through data comprised of reflective records of intervention delivery (*n* = 22), in-depth records of telephone implementation support meetings between research team members and co-researchers (*n* = 3), and in-depth evaluation meetings (*n* = 2). Data were qualitatively analysed using an NPT framework designed for intervention evaluation.

**Results:**

Clinical co-researchers readily incorporated the Family-Focused Support Conversation into their everyday work. The intervention changed family support from being solely patient-focused, providing information about patient needs, to family-focused, identifying family concerns about the significance and implications of discharge and facilitating family-focused care. Co-researchers reported an increase in family members’ involvement in discharge decisions and end-of-life care planning.

**Conclusion:**

The Family-Focused Support Conversation is a novel, evidenced-based and context specific intervention. Pilot implementation demonstrated the potential for the intervention to be used in acute hospitals to support family members during end-of-life care transitions. This subsequently informed a larger scale implementation study.

**Trial registration:**

n/a.

## Background

For many, the end-of-life is characterised by movement between hospital and home or nursing home, particularly in the last few weeks of life [1]. End-of-life care transitions can be practically and emotionally difficult for family members [2]. They signify the certainty of impending death [3] and evoke many uncertainties about the future, including care after discharge [4]**.** Thus, care transitions are a ‘critical time’ during which family members need additional support and information [5] and an opportunity for health and social care professionals to identify and respond to this need.

However, family members rarely receive the support and help they need during end-of-life discharge planning. Qualitative research reporting their experiences demonstrate a focus on organisational needs rather than family concerns [6, 7]. As a result, families lack the information and support they need to make informed decisions about their role in end-of-life care and how to harness family and community resources to provide and sustain care for their ill family member, once discharged [6].

Whilst there is a growing body of research about effective family caregiver support interventions at the end-of-life [8–28] none of this evidence specifically addresses how to provide family support during hospital admission or during the transition of care from hospital to home or nursing home at the end-of-life. Despite this, the interventional content is considered potentially transferable to other contexts, providing consideration is given to the specific needs of family caregivers [29], and their broader circumstances over time [30]. However, there is a paucity of research translating this evidence into realistic clinical applications [31, 32].

We therefore undertook a Participatory Learning and Action Research study to implement support for family members (those important to a dying person, irrespective of relationship), during the transition between hospital and home or nursing home, at the end-of-life. This paper provides an in-depth account of intervention development and pilot implementation, of a unique, brief intervention, the Family-Focused Support Conversation.

### Research approach

The study was informed by Normalization Process Theory (NPT), a structured and theoretically robust approach to understanding the factors that promote and inhibit implementation [33, 34]. NPT proposes that implementation is a dynamic interactive process, influenced by the social actions of those involved. This process is described by four constructs: coherence, sense making work; cognitive participation, relational work; collective action, operational work; and reflexive monitoring, appraising work.

NPT was integrated with a Participatory Learning and Action (PLA) Research approach, a combination previously demonstrated to positively influence the quality of intervention design and implementation, by ensuring inclusion of diverse sources of knowledge and expertise [35]. In this paper we describe three PLA cycles concerned with intervention development and pilot implementation. These cycles broadly followed an intervention development process described by Hawkins and colleagues [36] consisting of: evidence review; co-production of a conceptual framework and interventional structure and process; and pilot implementation.

## Methods

The aims were to:

- Critically review the research evidence base for supporting family members caring for a dying person, to identify the theoretical and therapeutic mechanisms of effective interventions (PLA cycle 1);
- Design a conceptual framework, theoretically modelled on the evidence review, and from this co-produce the structure and process of an intervention suitable for the acute hospital context (PLA cycle 2);
- Undertake a pilot implementation, to assess the potential for the intervention to be used in acute hospitals and understand whether it created unexpected work or disruption for family members and staff (PLA cycle 3).

PLA participants comprised:

- *Patient and public involvement [PPI] participants*, (*n* = 5) members of the public with experience of caring for a dying relative, recruited through National Institute of Health Research (NIHR) networks;
- *Clinical co-researchers* (*n* = 7), specialist nurses and occupational therapists, working in palliative and end-of-life care teams in 3 acute NHS Hospital Trusts in England (one in the South of England and two in the North). The teams were recruited through the National Nurse Consultant (Palliative Care) Group;
- *Social care experts* (*n* = 6), members of local and national carers groups and social workers with expertise in end-of-life care, recruited through palliative care networks.

All PLA participants were involved in the co-construction of the intervention in PLA cycle 2. Clinical co-researchers led implementation in PLA cycle 3. All PLA participants were involved in the interpretation of implementation results for PLA cycle 3..

### Evidence review (PLA cycle/aim 1)

The evidence review focused on research reporting interventions to provide family caregiver psychosocial support during palliative and end-of-life care. Psychosocial support was defined as support concerned with the emotional and relational wellbeing of family members [37].

There are a growing number of systematic reviews assessing psychosocial interventions for family caregivers during palliative and end-of-life care. However, systematic reviews rarely provide enough detail about interventions to allow implementation [38], but they do provide rigorous assessment of the methodological quality of studies. We therefore identified studies to review from these sources. Systematic reviews were identified from the meta-review by Thomas et al. [9], and from a search for systematic reviews published subsequently. All studies included by the review authors were assessed for relevance (Table 1). They were included if focused on caregiver support during palliative and end-of-life care, if graded by the systematic review authors as being of good methodological quality and if the intervention was amenable to delivery by hospital-based registered practitioners. Identified studies were analysed to identify the theoretical framework(s) underpinning the reported interventions and the interventional mechanisms identified by authors responsible for the reported therapeutic outcomes.

**Table 1:** Inclusion and exclusion criteria for evidence review

Included	Excluded
Studies focused on *caregiver* support	Studies not focused on caregiver support, for example, those focused on patient support
	Studies solely focused on symptom management
	Studies focused on service evaluation
Studies focused on *caregiver* support during *palliative or end of life care*	Studies focused on caregiver support before, during or after treatment, during survivorship or where prognosis was expected to be longer than 1 year
Studies graded by the systematic review authors, as good quality	Studies graded by the systematic review authors as weak quality
Studies of interventions *amenable to delivery by hospital-based, registered practitioners* (e.g. ward staff)	Studies focused on psychotherapeutic approaches e.g. cognitive behavioural therapy
	Studies using other therapeutic approaches such as art or music therapy or other focus such as training caregivers to use prompt questions in consultations

### Co-production of conceptual framework and intervention design (PLA cycle/aim 2)

The outcomes of the evidence review were synthesised into a conceptual framework from which the structure and process of the intervention was co-constructed with PLA participants. There is very little guidance about how to undertake this process in healthcare [39, 40] but we previously found participatory theatre techniques [41, 42] valuable in drawing out participants’ cultural, clinical and social knowledge to inform the translation and synthesis of evidence into healthcare processes [43]. We integrated these approaches in the following steps:

1. Workshop with PPIs, clinical co-researchers and social care experts: ‘theatre of language’ and ‘forum theatre’ [41] techniques were used to discuss the evidence and from this identify key principles for the conceptual framework and then map the identified intervention mechanisms onto to the structure of a typical clinical conversation [44].
2. The mapped clinical conversation was refined by developing conversational prompts through simulation and rehearsal of the intervention. The PI (SD) acted as clinician, an educational performative theatre expert as family member and the Senior Research Fellow (NC) as observer, providing reflective comments to guide simulation. The simulation was video-recorded and transcribed into a description of the interventional process.
3. The transcribed interventional process was discussed with PLA participants and mapped to a theoretical framework. This process resulted in a conceptual framework and intervention.

### Pilot implementation (PLA cycle/aim 3)

Following ethical and local site research governance approvals (REC ref.: 16/SC/0330) pilot implementation was undertaken over a three-month period by seven clinical co-researchers (described above), most of whom were involved in intervention development. In preparation, clinical co-researchers attended a half-day workshop where the intervention was reviewed, discussed and rehearsed. Given one of the purposes of pilot implementation was to assess potential disruption caused by implementation, each clinical co-researcher was asked to limit intervention provision to five interventions.

Data collection was informed by NPT and collected via reflective records of intervention delivery, detailed records of telephone conversations between the research team and clinical co-researchers discussing implementation progress and detailed records of evaluation meetings.

Data analysis was undertaken by three members of the research team (NC, SL, SD), following a Framework Analysis approach [45]. Data were initially coded, using the broad constructs of NPT, followed by a more detailed analysis, using NPT-generated questions designed by Murray et al. [46] as an analytical framework. A workshop to discuss interpretation of data analysis was held with PLA participants.

### Ethics

In addition to research ethics and site governance approvals required for implementation (REC ref.: 16/SC/0330), PLA raises ethical concerns about the location of ‘power’ in researcher and participant relationships and how this is managed to achieve the collaborative relationship intended [35]. Detail of how we approached these concerns is provided in supplementary information 1.

## Results

### Evidence review results (PLA cycle/aim1)

**Fig. 1:**
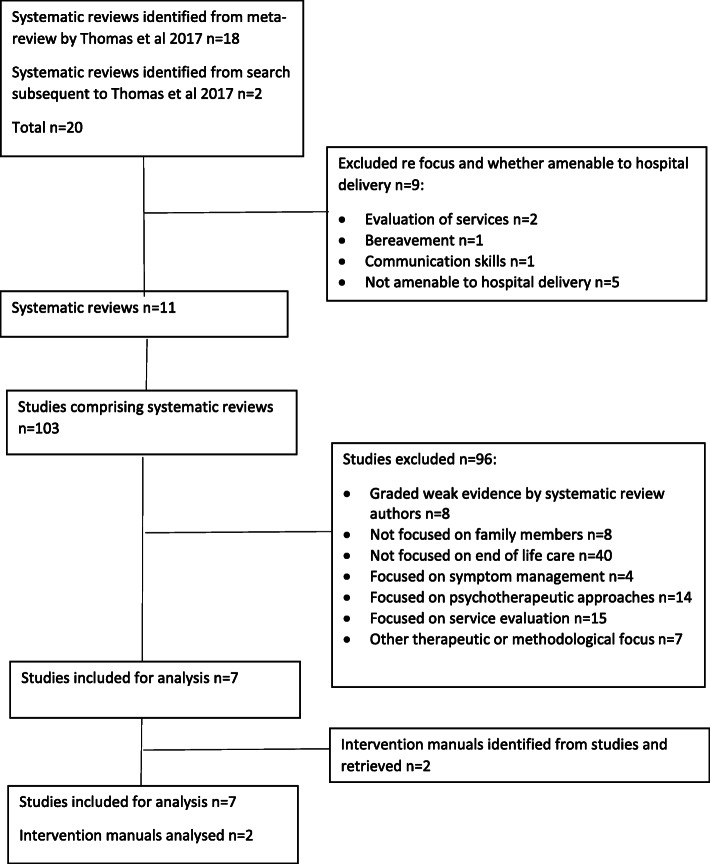
Evidence review process

Full details of the evidence review is provided in supplementary information 2. The process is summarised in Fig. 1.

A total of 20 systematic reviews were identified; 18 reviews graded as good or medium quality by Thomas et al. [9], focused on end-of-life care or cancer care [8, 10–26] and 2 systematic reviews published subsequently [27, 28]. Nine reviews were excluded because they focused on evaluation of services [10, 11], bereavement interventions [12], communication [13], or interventional approaches not amenable to hospital provision, such as art therapy [14], physical activity [15], web-based delivery [18] and mindfulness [27, 28]. The remaining 11 systematic reviews [8, 17–26] comprised 103 studies of which 96 were excluded.

The seven papers identified for analysis, related to 6 studies focused on psychosocial support for family members, during end-of-life care [47–53]. Two of these studies related to the FOCUS [49, 50] and COPE [51, 52] interventions and therefore manuals for these interventions were obtained from the authors.

Four studies explicitly stated the theoretical framework(s) adopted [47–50], in one it was implicit [50, 51] and in another not stated [53]. Five studies were underpinned by stress and coping frameworks derived from the work of Lazarus and Folkman and aimed to mitigate uncertainty, distress and carer burden, enhance family communication and increase access to resources [47–52]. One study also drew on Horowitz’s work [47], which emphasised the importance of meaning-making in adaptation to difficult life events (Table 2). Thus, collectively, the reviewed studies were theoretically modelled on stress, coping and meaning making, and focused on quality of life.

**Table 2:** Summary of review of intervention studies of family support during palliative and end of life care

Authors	Aim of intervention	Theoretical framework	Intervention processes				
			Information	Meaning	Resources	Problem solving	Self-care
Hudson et al. (2005) [48]	To enhance support and guidance for caregivers	Lazarus and Folkman’s transactional model of stress and coping	Information about typical aspects and common issues associated with caregiver role Provide opportunity to access information and provide basis for skill acquisition	Helping caregiver to construct meaning, normalising emotional reactions, encouraging them to see positive aspects of experience, offering spiritual guidance	Reinforcement role of p/c services and other services and providing strategies for involving family and friends	To provide options by offering caregivers an opportunity to identify issues and plan goals/strategies and advising caregivers of their rights	To promote self-care by encouraging caregivers to enhance their physical and mental health by taking time out, exercise, sleep, relaxation
Manne et al. (2004) [47]	Not specifically stated but aim was to evaluate impact of psychoeducational group intervention	Stress and coping theory (Lazarus, 1994) and social and cognitive processes of adaptation to difficult life events (Horowitz, 1986) – difficult events challenge preconceptions of world, must make sense of situation	Information about cancer and treatment and treatment side-effects Education about importance of open communication	Theoretically predicated on sense making Education about adaptive coping (finding meaning and benefit in experience) and about maladaptive coping (denial, avoidance)	Not reported	Skills training in communication	Not reported
McMillan et al. (2006) [51] McMillan and Small (2007) [52]	To increase caregivers QOL and sense of mastery in order to reduce caregiver burden and enhance caregiver coping	Not stated but implicitly stress and coping theory	Lay information	(optimism)	Not reported	Problem-solving coaching with four component – creativity, optimism, planning, information	Not reported
Northouse et al. (2005) [49]	To improve stress appraisal variables (appraisal of illness or caregiving, uncertainty, hopelessness), coping resources, symptom distress and QOL	Stress appraisal model from Lazarus and colleagues	Provision of information to reduce uncertainty	Encourage optimistic outlook	Not reported	Coping effectiveness Education about how to manage symptoms	Not reported
Northouse et al. (2007) [50]	To improve stress appraisal variables (appraisal of illness or caregiving, uncertainty, hopelessness), coping resources, symptom distress and QOL	Stress appraisal model from Lazarus and colleagues	Provision information to reduce uncertainty	Encourage optimistic outlook	Not reported	Coping effectiveness Education about how to manage symptoms	Not reported
Walsh et al. (2007) [53]	To provide increased support for people caring for patients receiving specialist palliative care	Not stated	Advice about patient care andphysical care needs	Advice about planning for future Advice about psychological health, relationships and social networks	Advice about health and social care providers	Not reported	Advice about time needed away from patient

The interventional processes identified from the review included the provision of information, sense-making, problem-solving, resource networking and self-care. All interventions were delivered over successive consultations, for example FOCUS and COPE were provided in three structured visits of at least 30 min each. There was insufficient detail in the reviewed studies to assess the effectiveness of proposed therapeutic mechanisms on outcomes. Therefore, in consultation with PLA social care experts and clinical co-researchers, we considered it prudent to adopt recommendations from Northouse and colleagues [17] to focus intervention processes on mitigating uncertainty and from Candy and colleagues [8] to at ‘*the very least healthcare practitioners should enquire about the concerns of family and friends caring for a loved one’* (p23) and incorporate information and problem-solving coaching processes, to buffer psychological distress.

### Conceptual framework and intervention structure and process (PLA cycle/aim2)

PLA participants stressed the importance of the intervention being equally applicable to any family member, irrespective of their role in future care, and deliverable as the opportunity arose. Use of a pre-existing intervention such as FOCUS or COPE was therefore considered impractical and inappropriate. Therefore, to ensure the intervention could be delivered flexibly, depending on family and clinical circumstances, we used a structured conversation design, a brief intervention design considered clinically feasible for provision of evidence-based psychosocial support [54].

In addition, PLA participants emphasised the importance of involving family members in end-of-life care decisions, recognising their knowledge of the ill person and ‘how they do things as a family’. Therefore, the intervention was theoretically modelled on Family Sense of Coherence [55]. Family Sense of Coherence is theoretically congruent with the stress, coping and adaptation theories underpinning the reviewed studies, but rather than focusing on ill health (distress) and quality of life, it focuses on family strengths, resilience (coherence) and salutogenesis (wellness). Coherence is influenced by whether life events are considered comprehensible, manageable and meaningful and congruent with ‘how we do things as a family’ [55]. Family Sense of Coherence was also theoretically consistent with the interventional processes of meaning-making, problem-solving and harnessing resources, identified in the evidence review.

Importantly, the meaningfulness component of FSC is considered key to family coherence [55]. Meaning focused approaches are particularly helpful in situations characterised by uncertainty [56] by helping to realign priorities and create or renew a sense of purpose [57]. This approach therefore suited end-of-life care transitions where family priorities move towards the care of a dying member, when there is limited time to make decisions about place of care and where there is uncertainty about care provision and the future. Thus, addressing uncertainty by focusing on meaningfulness is likely to strengthen family coherence. Family members with strong family coherence are likely to have positive caring experiences [58–60], and confidence in end-of-life care provision [61].

**Fig. 2:**
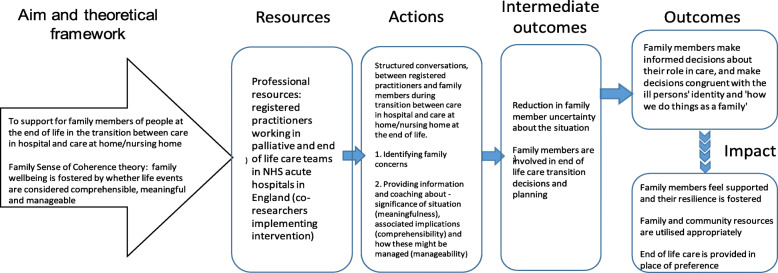
Family-Focused Support Conversation: Conceptual Framework and Logic Diagram

Consequently, the internal logic of the resulting conceptual framework proposed that family members’ uncertainties about end-of-life care could be reduced by identifying and addressing their concerns, by providing information and coaching problem-solving. As a result, family members would make informed decisions about their role in care and harness family and community resources. Thus, the intervention would foster family coherence (resilience) (Fig. 2).

**Fig. 3:**
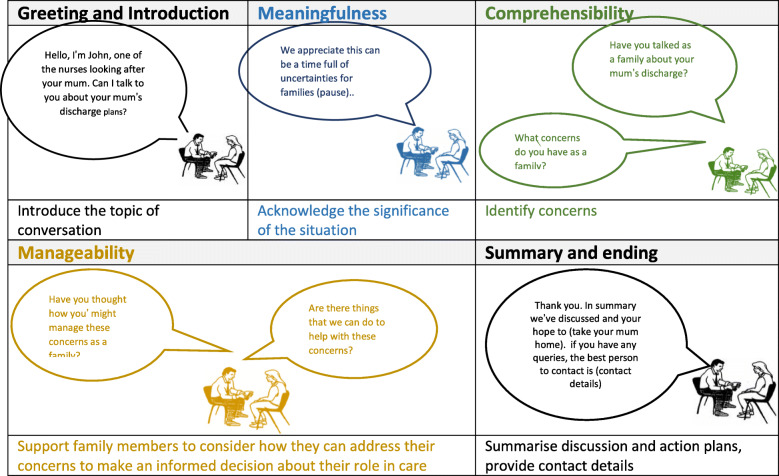
Family-Focused Support Conversation – process

The structure and process of the intervention, resulting from the participatory theatre workshops, are outlined in Table 3 and Fig. 3. This description includes a revision made by clinical co-researchers during implementation training, to use an empathetic phrase at the beginning of the intervention to orientate participants to the family focus of the conversation, followed by a pause. This combination of empathetic statement and pause, increases the likelihood that concerns are raised and addressed in clinical consultations [62].

**Table 3:** Structure and process of intervention and underpinning theoretical framework

Conversation sequence (Duke and Bailey, 2008)	Intervention Process	Conversational Prompts	Therapeutic purpose	Theoretical framework – Family Sense of Coherence
**Greeting and introduction of self to family member**				
**Focus of conversation**	Empathetic statement followed by a pause	We wanted this opportunity to talk to you as a family about the care needed by [relative] and the concerns you may have. We appreciate this can be a difficult time for families (pause)	To understand the meaning and significance of the care transition to the family	Meaningfulness: How the family is making sense of the situation. Meaningfulness is the motivational dimension of FOC, concerned with the extent to which life’s problems are worth committing to, as challenges as opposed to burdens – a desire to resolve difficulties and a willingness to invest energy to get through a stressful situation (Antonovsky, 1991:41)
**The story so far**	Asking about concerns	Have you talked as a family about the care of [relative] Have these discussions raised any concerns for your family?	To determine family concerns about the care transition and potential implications	Comprehensibility: the family’s understanding of the situation (implications of the care transition for family members) Comprehensibility is the cognitive dimension of FSC – the extent to which internal and external stimuli are understandable and the willingness and ability to organise and sort information (Antonovsky, 1991:39)
**How this might change or be thought about differently**	Information and Problem-solving coaching	Have you had any thoughts as a family about how you might manage those concerns? Are there things we could help you with?	To facilitate family involvement in care planning, to coach solutions to the implications raised, and help family members harness family and community resources in an appropriate plan	Manageability: The family’s ability to manage the implications of the situation and harness their resources Manageability is the instrumental or behavioural dimension of FSC – the degree to which you feel you have the resources to meet challenges and the willingness to solve problems of challenges faced (Antonovsky 1991:40)
**Plans for next steps**				
**Concluding and ending**

### Implementation results (PLA cycle/aim3)

Data consisted of *n* = 22 reflective records of intervention delivery (site 1: *n* = 5; site 2: *n* = 10; site 3: *n* = 7), *n* = 3 records of discussion of implementation progress and *n* = 2 records of evaluation meetings.

Table 4 provides a summary of analysis, constructed from data coded against NPT-generated questions for intervention evaluation [46]. The table provides the source of data quoted in italics below. All quotes were provided by clinical co-researchers - specialist nurses and occupational therapists in palliative care..

**Table 4:** Implementation results – summary of analysis using NPT generated questions for interventions (Murray et al., 2010)

NPT-guided questions (Murray et al, 2010).	Clinical Co-researcher evaluation
**Coherence - Meaning and sense-making by participants**	
Did co-researchers find the intervention easy to describe?	The intervention was more readily understood by those involved in the intervention development. Those not involved needed to see the intervention delivered or to deliver it themselves, ‘*to get it’* (evaluation meeting B). Comprehension of the intervention was enhanced by thinking through how to phrase the intervention, so that it was congruent with their clinical style and patient population, and with experience of delivery (evaluation meetings A and B).
Was it clearly distinct from other interventions?	Initially some co-researchers found it difficult to differentiate the intervention from their normal practice but as they delivered the intervention they described a subtle shift in their understanding from something professionally focused and ‘*done to families*’ to being family-centred (evaluation meeting B), ‘*flipping the conversation to focus on the family (the meaning for the family’* and their concerns (evaluation meeting B). Co-researchers also discriminated between the intervention and discharge ‘*it’s not about discharge itself*’ or ‘*bits of kit*’ or ‘*a checklist’* (evaluation meeting B)
Did it have a clear purpose for all those involved?	Co-researchers were self-selected and likely to already value and be motivated to provide family care.
Did participants have a shared sense of its purpose?	Co-researches developed a shared purpose of the intervention by discussing their experience of intervention delivery as a team, often immediately after an intervention or when encouraged by the implementation lead for the team
What benefits did the intervention bring and to whom?	Co-researches described they had ‘*pulled apart their practice*’ by delivering the intervention, which improved their practice by approaching family support from a ‘*different angle’*, ie from the family’s perspective, to ‘*comprehend the impact of words and phrases on family members*’ (evaluation meeting B).
Were these benefits likely to be valued by family members?	Co-researchers described family benefits in terms of having the difficulty of the situation and their care acknowledged and providing time for them to think about the support they might need. They also thought that it helped family members with decision-making and problems-solving – gives family a voice in planning and helps them to work together to provide care.
**Cognitive participation - commitment and engagement by participants**	
Did co-researchers and family members think the intervention a good idea?	Yes, see above. However, co-researchers varied in their opinion about when the intervention was a good idea. Some delivered the intervention whenever they spoke to a family member about discharge care plans, irrespective of circumstances, whereas others discriminated depending on the circumstances, choosing not to use the intervention when, for example, there were disagreements between family members about where care might be provided. Some co-researchers also described circumstances where they started to deliver the intervention but then stopped, because of the family member’s response – being unduly distressed or revealing a change in discharge plan.
Did they see the point of the intervention easily?	Yes, co-researchers valued family-focused care, but see above how their perception of the intervention changed from being perceived as like normal practice to being distinct.
Did they invest time, energy and work in it?	One person in each of the teams took a leadership role to explain the intervention to other members of the palliative care team, ward teams, discharge liaison leads and community teams. Cascading this information was influenced by the dynamics of the team but all teams developed strategies to overcome encountered difficulties. Some co-researchers described how they became increasingly invested in the intervention with the more experience they had of delivering it – the benefits becoming increasingly clear with increased delivery.
**Collective action - How will the work of the intervention affect user groups?**	
How did the intervention affect the work of co-researchers?	Some co-researchers initially thought that the intervention would take more time than normal care and explained that it felt ‘*clunky*’ at first (evaluation meeting A). However, as they became experienced, they found that the intervention flowed and conversations became more focused - it helped to identify family concerns quicker than usual practice and overall saved time.
Did it promote or impede their work?	Some evidence was provided that the intervention promoted co-researchers satisfaction with their work: ‘*I actually quite enjoy discharge now*’; ‘*discharge feels less onerous now*’ (evaluation meeting B) Some co-researchers described how the intervention had changed what might be expected of families, enhanced their appreciation of families and what they are doing in end of life care transitions. Some co-researchers described how the intervention had enhanced all of their work, integrating within their practice the habit of asking both patients and family members ‘what are your concerns’.
What effect did it have on their consultations?	Co-researchers needed to deliver the intervention a few times before it felt a natural process. Initially co-researchers used the intervention in consultations specifically directed at discharge planning but as some co-researchers became confident in its use, they incorporated it into other conversations.
Did co-researchers require extensive training before they could use it?	Co-researchers were experienced practitioners in palliative care and had considerable expertise in communication skills and service development. Training consisted on a half day explanation and demonstration of the intervention, followed by team-based discussion, rehearsal and reflection.
How compatible was it with existing work practices?	The intervention was considered compatible with existing work practices but assessment about the appropriateness of delivery was influenced by contextual challenges such as resource constraints. Some co-researchers explained how organisational pressures, and the resulting increase in their workload ‘*prevented meaningful conversations with families*’ (evaluation meeting B). Some also described how these pressures resulted in insufficient time to set up a meeting with family members before discharge or where family conversations had already taken place by other staff.
What impact did it have on the division of labour, power and responsibility between different professional groups?	Implementation raised the way in which end of life care work is distributed in hospitals and how this influences access to family members and the provision of support. Implementation was influenced by the division of labour between specialist and ward teams. Specialist and ward teams typically have working arrangements about each other’s roles in discharge. Some co-researchers were concerned the intervention would disrupt these agreements and give a contradictory message to colleagues about their role in discharge and family support. During this pilot phase, co-researchers tended to focus the intervention on situations where discharge conversations had yet to take place with family members and were therefore likely not to occur when death was imminent.
Did it fit with the overall goals and activity of the organization?	The intervention was considered an alternative for measuring effectiveness of specialist palliative care – a process by which quality standards could be achieved and demonstrated.
**Reflexive monitoring - Participants reflection and appraisal of the intervention**	
How did co-researchers perceive the intervention once it has been in use for a while?	Several co-researchers described how the intervention had ‘changed their practice’ and provided satisfaction in relation to discharge planning and family support.
Was it perceived as advantageous for family members and staff?	Co-researches described how the intervention ‘makes a difference to families’ and has ‘outcomes for the family in terms of their resilience’. Some gave examples of benefit: • helping a wife to express concerns that she would not be allowed to take her husband home to die and thus enabling this to happen; • helping a wife to understand the care that would be available at night and to problem-solve how to manage this with her children’s help; • a cousin of a patient who received the intervention reported to the co-researcher ‘you are the first person to ask what are my concerns’, explaining that his concerns had been overlooked up until that point
Are the effects of the intervention clear?	Co-researchers considered that the intervention results in families gaining better psychological support compared to normal practice. Consequently, co-researchers were beginning to think about involving families earlier in conversations about care transitions.
Can the intervention be adapted or improved?	Some co-researchers reported altering the order in which the interventional components were delivered, for example delaying the acknowledgement statement (meaningfulness) until later in the intervention if it felt ‘flippant’ to use at the beginning. They also reported that comprehensibility and manageability components tended to flow into each other. Co-researchers also described how they occasionally delivered the intervention over more than one consultation, sometimes ending the intervention after providing information which addressed family concerns and asking them to talk together before coming back and delivering the last component, focused on problem-solving and making an action plan. They also discussed delivering the intervention by telephone.

### The potential for the intervention to be used in acute hospitals

All clinical co-researchers implemented the intervention. Some needed to see the intervention delivered or to deliver it themselves, ‘*to get it*’. Most needed to practice how to phrase the interventional prompts so that the intervention ‘*flowed’* and was consistent with their communication style and patient population. Some approached implementation by delivering the intervention whenever they spoke to a family member about discharge care plans, irrespective of circumstances. Others initially selected opportunities to use the intervention, starting with more straightforward discharge situations. As co-researchers gained confidence, the intervention was incorporated into practice, irrespective of the complexity of discharge.

Some clinical co-researchers were initially concerned the intervention would take more time than normal care but found it enabled focused discussions and quickly identified family concerns, and thereby saved time. As they gained confidence they moved backwards and forwards between the comprehensibility and manageability components of the intervention to address family concerns. Nevertheless, implementation was influenced by contextual challenges such as resource constraints and organisational pressures on their work, for example when organisational pressures resulted in ‘*late referrals*’ and insufficient time to meet with family members before discharge. Some managed these pressures by splitting delivery over successive consultations, introducing the focus of the intervention (meaningfulness) in the first meeting (in person or by telephone) and completing delivery at a second meeting.

### Did the intervention create unexpected work or disruption for family members and staff?

Co-researchers described how the intervention disrupted and ‘*pulled apart their practice*’ by ‘*flipping conversations to focus on the family*’. They described a shift in their understanding of family support as something professionally focused and ‘*done to families*’ to practice centred on ‘*the meaning for the family and their concerns*’. They also discriminated the intervention from ‘*discharge itself*’ or ‘*bits of kit*’ or ‘*a checklist*’. They reported a deeper understanding and respect for family care and increased satisfaction with discharge work and provided examples of how the intervention enhanced family care (see Table 4).

Implementation was also influenced by the local working arrangements for discharge between specialist and ward teams. When discharge was considered a ward team responsibility, clinical co-researchers were worried the intervention would disrupt these arrangements and create ambiguity about their role. Some clinical co-researchers therefore restricted use of the intervention to occasions when discharge conversations had been commenced with family members by ward staff. Others used the intervention to clarify concerns family members had previously raised with ward staff or by delivering the intervention in collaboration with ward staff.

## Discussion

In the three PLA cycles reported above, we co-constructed and implemented the Family-Focused Support Conversation. This structured conversation is underpinned by a robust review of research evidence, family members’ experiences and clinical expertise, and theoretically informed by Family Sense of Coherence, a theory which emphasises family strengths and resilience.

The evidence review identified the importance of modelling interventions for family support on uncertainty [17], and therefore asking family members about their concerns [8]. Consistent with other reviews we found a lack of interventions adopting a family centred or family systems approach [2] and a lack of translation of research evidence into feasible clinical interventions [31, 32]. Similarly, with respect to intervention reporting, we found inconsistent detail about the theoretical frameworks underpinning interventions [63] and insufficient evidence about the effectiveness of intervention mechanisms on outcomes [8, 9, 29, 63].

Pilot implementation demonstrated the potential for the Family-Focused Support Conversation to be adopted in acute hospitals. Clinical co-researchers made sense of the intervention (coherence), actively engaged in implementation (cognitive participation), worked together to provide the intervention (collective action) and reflected on its benefits and costs (reflexive monitoring).

The reported shift in co-researchers’ practice, as a consequence of implementing the intervention, is notable. All co-researchers valued family support as part of their palliative care role but described how their practice was subject to the organisational constraints and priorities experienced by most hospital practitioners [6, 7]. Thus, some described how previously they focused on discussing discharge arrangements with family members rather than family concerns. The intervention enabled a shift in focus to family concerns about the meaning, significance and implications of discharge, and how family caregiving might be managed.

In part this shift occurred because of the strong coherence between the value co-researchers placed on family support and the purpose and design of the intervention. Co-construction meant co-researchers’ values were integrated within the design of the intervention. However, because these values were combined with those of family members, social care experts and research evidence, and integrated through theoretical modelling and clinical simulation, the intervention provided a structured communication process by which the inherent tensions in purpose between hospital organisational priorities and family support could be reconciled [64]. Thus, the structured conversation weaves intervention components in a clinical conversation and provides a clinically feasible [54] means of providing family support and to optimizing family outcomes [64].

However, the results also demonstrated how organisational discharge priorities were embedded within the distribution of work between practitioners. Where ward teams had responsibility for end of life discharge, implementation threatened to disrupt previously negotiated roles between ward and specialist teams and co-researchers were concerned about creating role ambiguity. In these instances, the noted shift in practice was also due to the negotiation strategies used by co-researchers to implement the intervention, and sustain their relationships with ward teams, without causing ambiguity about their respective roles. However, it is important to recognise this account does not include the impact of implementation on ward staff or their experience of the negotiation strategies used by co-researchers.

Our decision not to involve ward teams in the study was a consequence of the role we wished co-researchers to play in the reported phases of the study. We considered it important for co-researchers to have palliative care expertise and communication, reflective and service development skills to influence the design of the intervention and lead implementation. The rich knowledge generated by co-researchers, discussed above, is testament to these skills and to their adaptation to a research leadership role, something which markedly contrasted with most co-researchers’ previous experiences of research. This reversal of roles reinforced the importance of a research team having pedagogic skills to support co-researchers in this leadership role.

The knowledge generated in the three PLA cycles described in this paper was used to further implement the Family-Focused Support Conversation in 12 NHS acute hospitals in England and to qualitatively evaluate its usability, acceptability and accessibility and will be reported elsewhere.

## Conclusion

Discharge from hospital at the end-of-life is complex and organisational priorities often result in family members lacking the support needed to make informed decisions about their role in end-of-life care on discharge. Through a process of co-construction, we designed an evidence-based structured conversation, the Family-Focused Support Conversation. Pilot implementation demonstrated the intervention has the potential to be adopted in acute hospitals, and addresses family concerns about the meaning and significance of discharge, implications about end-of-life care needs and how family caregiving can be managed and enhanced.

## Supplementary information

**Additional file 1: Supplementary information 1.** Information for PLA participants – PPI colleagues, clinical co-researchers and health and social care experts.

**Additional file 2: Supplementary information 2.** evidence review.

**Additional file 3: Supplementary information 3.** Description of Family-Focused Support Conversation using TIDieR checklist (Hoffman et al, [65]).

## Data Availability

All data generated or analysed for this study are included in this published article [and its supplementary information files].

## Abbreviations

FSC: Family Sense of Coherence
PLA: Participatory Learning and Action (Research)
NPT: Normalization Process Theory
