# Supplementary material for: Co-construction of the family-focused support conversation: a participatory learning and action research study to implement support for family members whose relatives are being discharged for end-of-life care at home or in a nursing home
Source: BMC Palliat Care. 2020 Sep 21;19:146. doi: 10.1186/s12904-020-00647-5 (PMC7507823; doi:10.1186/s12904-020-00647-5)
Supplement: Supplementary file 1 — Additional file 1: Supplementary information 1. Information for PLA participants – PPI colleagues, clinical co-researchers and health and social care experts. [file 12904_2020_647_MOESM1_ESM.pdf]

Supplementary information 1: Information for PLA participants – PPI colleagues, clinical co-researchers and health and social care experts

Supporting family caregivers in the transition from hospital to their relatives preferred place for end of life care

Thank you for agreeing to take part in this research to support family caregivers in the work that they do during discharge from hospital to home for end of life care.

Background to the research approach

The research is designed using Participatory Learning and Action (PLA) methodology, conducted in developmental cycles.

We have chosen this approach because we believe that it is not possible to achieve our aim of implementing family caregiver support without:

a. Valuing practitioners' clinical wisdom and knowledge of the context in which this takes place
b. Integrating this knowledge with research knowledge

We also believe that to achieve this aim we need to work in equal partnership with you, designing the work that we do together, fully accrediting your contributions as co-researchers and ensuring that you have full access to anything that is produced.

One of the challenges reported by PLA co-researchers is that they can be unsure about what is expected of them. We hope the information below will help to address this challenge.

Working with you as co-researchers

As co-researchers we will work with you to identify your knowledge and contribution to the products of the study. We will ask you to review these products as the research progresses.

The work is developmental so this means we will not fully know what work will be involved in each PLA cycle until it is designed in the previous cycle. However we will discuss with you whether you are happy to continue or renegotiate your involvement at the end of each cycle.

We hope that you will work with us initially for the first three PLA cycles, which involve:

- Designing a support intervention* (PLA cycle 1)
- Designing the governance processes needed to support the intervention* (such as training) (PLA cycle 2)
- Trying the intervention* out in practice (PLA cycle 3 and 4)
- Helping us to evaluate the implementation of the intervention* in practice (PLA cycle 5)
  *by intervention we mean the process by which support is provided and this is likely to be through a relatively brief clinical conversation and follow up information.

The background work of each PLA cycle will be done by the research team so that the expectations of your contribution can be identified and negotiated before you commit to doing any work.

We will also reduce the impact of the research governance processes on you and your practice. The research ethics and local research governance processes will be conducted by the research team, keeping you fully informed of the permissions we are seeking, the governance processes we are following and any implications these may have for you.

We want this research to have a positive impact in enhancing the support of family caregivers but not at the expense of increasing your workload or causing additional difficulties. So we are equally interested in you helping us to understand why some things are difficult or not possible in practice and your help in helping us to construct something that is realistic and pragmatic.

We also want this work to help other practitioners too. We are therefore planning to design toolkits and write policy templates that describe how to provide support and the resources needed. We will publish these on a project website so that they can be publically available as well in academic papers and conference papers. We will invite you to comment on the preparation of these resources and accredit you as authors.

Detailed information about participation in each PLA Cycle

The PLA cycles in the study are underpinned by a sociological theory of implementation, Normative Process Theory (NPT). Each cycle relates to central construct of NPT, and describes the work of implementation.

PLA Cycle 1: Designing a support intervention

PLA cycle 1 is concerned with Coherence. This is sense making work.

In this cycle we will work to:

1. Understand the work that family caregivers do to facilitate discharge from hospital at the EoL. We know from experience that this includes things like liaising with other family members and hospital staff, being a spokesperson for their relative, re-organising furniture at home, receiving equipment, viewing nursing homes etc.
    - *What we have done so far*: we have undertaken a literature review and have planned a PPI event to find out more about this work with family caregivers
    - *How we hope you may contribute*: your knowledge of what work family caregivers do to facilitate discharge
2. Understand how best to provide support for family caregivers in the work that they do to facilitate discharge at the EoL given the pressures of acute hospitals and the aims, objectives and benefits of this practice.
    - *What we have done so far*: we have interrogated the literature and identified key principles that might form the key ingredients of a support conversation. We have also sought professional opinion from social work colleagues about the challenges of implementing family caregiver support.
    - *What we hope you may contribute*: what you think about the key principles and how they can be integrated into practice
3. Understand how the 'new' practice differs from how caregivers are currently supported – what makes it distinctive;
    - *What we have done so far*: we have examined existing interventions and identified their characteristics and why they are not fit for our purpose
    - *What we hope you may contribute*: how to portray the practice we co-design in a way that makes its difference explicit
4. Understand how practitioners might need to organise their work in order to provide the 'new' practice (who will provide the support, when and what things are needed for this to happen).
    - *What we have done so far*: we have begun to synthesise discharge policies to identify who does what and when
    - *What we hope you may contribute:* how things work in reality;
5. Understand what the benefits might be for family members, the person they are caring for and practitioners providing the support
    - *What we have done so far*: we have gathered information from the literature of what is of help to family members
    - *What we hope you may contribute:* your perspective on the challenges of providing support and what needs to be prioritised in an intervention to achieve a balance between benefit and the practice challenges.

PLA cycle 2: Designing how the intervention will work in practice

PLA cycle 2 is concerned with cognitive participation. This is to do with the relationship work needed for the intervention to work in practice – such as designing training materials, informational materials about the intervention and service specification about who will be delivering the intervention and when and how.

In this cycle we will work to:

1. Identify who will be using the intervention in practice. We know from our discussions with you and from our clinical experience that conversations with family members are undertaken by many practitioners and that these conversations are often opportune rather planned and may not be at a precise time in the discharge process. From talking to you we also recognise that such conversations require skill and sensitivity and that they can be difficult because of the end of life context of this work and the unpredictability of discharge planning
    - *What we have done so far*: we have identified that in the first instance the intervention should be delivered by palliative and end of life care team members, in participating teams
    - *How we hope you may contribute*: identifying other members of the team who might be involved; helping us to draw up a service specification which identifies who will be delivering the intervention and the skills required
2. Identify how and when the intervention will be used
    - *What we have done so far*: designed intervention criteria that describe the intervention – when and how it will be delivered
    - *How we hope you may contribute*: working with team colleagues to work out how best these criteria can be fitted into your everyday work
3. Design information and education about the intervention for practitioners – a training package
    - *What we have done so far*: we have undertaken a thorough literature review which explains the basis of the intervention and why it is important; we have details about the process of the intervention; we have identified an education framework that could guide the construction of training package
    - *How we hope you may contribute*: we would like some help to construct the training package so that the theoretical basis of the intervention is clinically sensitive and easily and quickly understandable
4. Design clinical governance processes that enable everyone to know who has received the intervention and how the intervention delivery went
    - *What we have done so far*: we have designed a anonymous record sheet as an aide memoire for recording the process and outcome of the intervention
    - *How we hope you may contribute*: we need some help with ensuring that team members are trained to use the intervention and that the team has a process for monitoring the use of the intervention as part of clinical governance processes (this will not be accessed by the research team or be part of research data).

PLA cycle 3 and 4

PLA cycle 3 is concerned with collective action. This is about putting operationalising the intervention. We have divided this into two parts – the first part (PLA cycle 3) is concerned with piloting the intervention in three settings and in the second part (PLA cycle 4) rolling the intervention out into 7 other NHS Trusts.

In PLA cycle 3 and 4 we will work to:

1. Understand whether and how the interventional components fit into everyday clinical conversations;
    - *What we have done so far*: we have modelled the intervention components onto possible clinical questions that could be asked of family members
    - *How we hope you may contribute*: to try out these questions in practice and reflect on what worked and do not work and record this reflections very briefly on an anonymous record sheet, which we will use to prompt a reflective evaluation at the end of this phase of the study
2. Understand what the clinical governance issues are related to the intervention – what things come up in practice related to the intervention and how these are best managed;
    - *What we have done so far*: IN PLA cycle 2 we will identify with you how you will manage your accountability in relation to providing the intervention (eg record keeping, provision of information to colleagues and to patients and family members)
    - *What we hope you may contribute*: we need to know whether these processes are working, and whether any additional processes are necessary to support the intervention
3. Understand who is delivering the intervention and what influences this;
    - *What we have done so far*: In PLA cycle 2 we will identify with you who will be delivering the intervention and the skills and resources they need to do so
    - *What we hope you may contribute*: knowledge of how the work of supporting family members is allocated in the team and whether this evenly distributed or whether decisions are made based on particular needs and skill mix
4. Understand the resources that are needed to deliver the intervention;
    - *What we have done so far*: In PLA cycle 2 we will work with you to design supporting resources such as training package and information sheets
    - *What we hope you may contribute:* knowledge about whether these resources are fit for purpose, and how they are being used and whether additional resources are needed.

PLA cycle 5

PLA cycle 5 is concerned with reflexive monitoring. This is appraisal work and is about evaluating the usefulness and practicality of the intervention. By the end of this cycle we hope to have co-produced a service model with you, describing the intervention in practice and toolkits to help other practitioners implement family caregiver support.

In this cycle we will work to:

1. Determine how effective and useful the intervention is to family members.
    - *What we have done so far*: we have designed an evaluation plan and are seeking ethical approval to invite family caregivers who have received the intervention to complete a short evaluation and consider participation in an interview
    - *How we hope you may contribute*: we would like to ask you to give a sealed pack to family members who have received the intervention. We will prepare the packs for you. The packs will contain the short evaluation questionnaire, and an invitation to participate in an interview, along with a participant information sheet and a return slip and self-addressed envelope (addressed to the research team)
2. Determine what you think of the intervention individually and as a team.
    - *What we have done so far*: In PLA cycle 2 we will have designed with you a record sheet to note your reflections of delivering the intervention
    - *How we hope you may contribute*: we would like you to participate in a discussion about the intervention and its implementation, drawing on the record sheets to prompt your reflections, and co-construct with us an evaluation of your experience
3. Assess any changes or modifications needed and produce a service model and supporting toolkits
    - *What we have done so far*: We will analyse the data collected from teams involved with the implementation and from family caregivers and prepare a summary for discussion with you
    - *How we hope you may contribute*: we hope you will help us to co-construct an evaluation report of the study and refine any toolkits produced to support intervention by other teams.

Contact us:

Dr Natasha Campling, SSCR Senior Research Fellow, School of Health Sciences, University of Southampton, SO17 1BJ. n.c.campling@soton.ac.uk

Dr Sue Duke, Associate Professor, School of Health Sciences, University of Southampton, SO17 1BJ. sd11@soton.ac.uk
