# Supplementary material for: Co-construction of the family-focused support conversation: a participatory learning and action research study to implement support for family members whose relatives are being discharged for end-of-life care at home or in a nursing home
Source: BMC Palliat Care. 2020 Sep 21;19:146. doi: 10.1186/s12904-020-00647-5 (PMC7507823; doi:10.1186/s12904-020-00647-5)
Supplement: Supplementary file 2 — Additional file 2: Supplementary information 2. evidence review. [file 12904_2020_647_MOESM2_ESM.pdf]

Supplementary information 2: evidence review

Contents | Page

1: Systematic reviews identified	
1a: Identified by Thomas et al (2017) in meta-review	2
1b: Identified in literature search	3
2. Systematic reviews consulted	4
3. Summary of studies identified from systematic reviews	7
4. Detailed summary of studies included in each systematic review	11
Northouse et al 2010	11
Regan et al 2012	14
Waldron et al 2013	17
Candy et al 2011	18
Nevis 2014	19
Harding et al 2012	20
Chambers et al 2011	23
Caress et al 2009	24
Ussher et al 2009	26
Glasdam et al 2010	28
Hudson et al 2010	31
6. Analysis of FOCUS and COPE from intervention manuals	33
5. Analysis of included studies	36

1a) Systematic reviews Identified in meta-review by Thomas et al (2017)

**Reviews considered good methodological quality by Thomas et al (2017)***	
**Systematic reviews focused on interventions for caregivers of people with cancer**	**Systematic reviews focused on interventions for caregivers of people at the end of life with various conditions**
Lang and Lim (2014)	Candy et al (2011)
Northouse et al (2010)	Gomes et al (2014)
Regan et al (2012)	Nevis (2014)
Waldron et al (2013)	
**Reviews considered of medium methodological quality by Thomas et al (2017)**	
Harding et al (2012)	Glasdam et al (2010)
Chambers et al (2011)	Hudson et al (2010)
Caress et al (2009)	Loi et al (2014)
Ussher et al (2009)	Pottie et al (2014)
Gauthier and Gagliese (2012)	Walczak et al (2014)
Kaltenbaugh et al (2015)	

*Thomas et al (2014) graded the systematic views using the criteria in the box below:

Is there a well-defined question?
Is there a defined search strategy?
Are inclusion/exclusion criteria stated?
Are study designs and number of studies clearly stated?
Have the primary studies been quality assessed?
Have the studies been appropriately synthesised?
Has more than one person been involved at each stage of the review process? The criteria were scored as follows: yes=1; in part=0.5; no or not stated=0.
High scoring reviews (i.e. those reviews that scored 4 and over) went forward for full data extraction for the meta-review. Only brief summary information was extracted from reviews of lower quality (i.e. those scoring less than 4).

1b Systematic reviews identified from search

Search details	CINHAL plus	Medline	APA PsycINFO	Totals
Search terms<br>Systematic or meta-analysis or literature review +<br>End of life care or palliative care or death or dying or terminally ill +<br>Caregivers or family members or relatives or informal caregivers +<br>Interventions	n=32	n=68	n-0	
Limiters				
Publication after January 2016 (since Thomas et al meta-review search)<br>All adults<br>Peer reviewed<br>Abstract available				
Systematic reviews identified as potentially relevant	n=1[a]	n=1[b]		n=2<br>Jaffray et al 2016 [a]<br>Lui et al 2018[b]

2. Systematic reviews searched for studies to review

Review	Focus of review	No of studies included in review	Review searched?	Justification of inclusion/exclusion	Number of studies identified for analysis
Lang and Lim (2014)	Systematic review of the effects of art therapy for family caregivers of cancer patients	N=2	No	Studies focused on art therapy	0
Northouse et al (2010)	Meta-analysis of RCTs of interventions for family caregivers with cancer 1983-2009	N=29	Yes	Although focused on cancer, some of the studies related to end of life care	6
Regan et al (2012)	Systematic review of couple-based interventions for cancer	N=23	Yes	Although focused on couples' therapy and cancer, some papers included people at the end of life in the study sample	3
Waldron et al (2013)	Systematic review of RCTs psychosocial interventions where at least one outcome was QOL	N=6	Yes	Although focused on cancer, some of the included papers were related to palliative and end of life care	4
Candy et al (2011)	Systematic review of RCT of interventions to support friend or relative in terminal illness	N=11	Yes	Reasons for excluding n=6 studies p18	5
Gomes et al (2014)	Systematic review of palliative care home care	N=23	No	Focus of review on palliative care home care vs usual care.	0
Nevis (2014)	Evidenced based review of educational interventions in palliative care	N=6	Yes	Reasons for excluding n=4 studies p19	2
Harding et al (2012)	Updated systematic review of how informal caregivers in cancer and palliative care can be supported	N=33	Yes	Reasons for excluding n=29 studies p20	4
Chambers et al (2011)	Systematic review of psychosocial interventions for men with prostate cancer and their partners	N=21	Yes	Studies included to address caregiver support reviewed n=5 see p21	2

Caress et al (2009)	Narrative review of interventions to support family carers who provide physical care to family members with cancer	N=19	Yes	Authors included studies which were not exclusively focused on physical care, so their papers included in their review were assessed for relevance to end of life care family support. The n=10 papers identified as focused on managing symptoms were not reviewed	4
Ussher et al (2009)	Systematic review of psychosocial interventions for informal carers of cancer patients	N=23	Yes	Studies graded as weak evidence not reviewed (n=6) – p26	3
Gauthier and Gagliese (2012)	Systematic review of bereavement interventions, end of life cancer care and spousal well-being		No	Focused on bereavement interventions and outcomes	
Kaltenbaugh et al (2015)	Systematic review of web-based interventions to support caregivers of patients with cancer		No	Focused on web-based interventions	
Glasdam et al (2010)	Systematic review of interventions developed for carers of people with cancer, stroke, diabetes, cardiovascular disease.	N=32	Yes	Reasons for excluding n=31 studies p28	1
Hudson et al 2010	Systematic review of psychosocial interventions for family carers of palliative care patients	N=13	Yes	Reasons for excluding n=12 studies p31	3
Loi et al 2014	Systematic review of the psychological impact of physical activity on caregivers		No	Focused on physical activity	
Pottie et al 2014	Synthesis of 58 papers reporting caregivers' satisfaction with hospice services and impact of services on caregivers psychological well-being		No	Focused on hospice services	
Walczak et al 2014	Systematic review of end of life communication interventions – focus on communication about diagnosis and		No	Focused on communication of diagnosis and prognosis	

	prognosis and on changing an aspect of communication				
Applebaum and Breitbart 2013	Systematic review to determine state of science of interventions for cancer caregivers	N=49 interventions	No	Focused on intervention design. All studies reviewed by authors included in other reviews apart from Bowman et al 2009, which was excluded from this evidence review because study outcomes were not reported.	
Jaffray et al 2016	Systematic review of effectiveness of mindfulness for caregivers in palliative care		No	Focused on mindfulness	
Liu et al 2018	Systematic review of mindfulness-based stress reduction for caregivers of people with dementia		No	Focused on mindfulness	
Total number of reviews mined for relevant studies<br>Number of papers reviewed identified from systematic reviews		11<br>103		Number of studies identified for analysis<br>After duplications removed	37<br>**7**

3. Summary of papers included in evidence review

Study	Included	Excluded	Reason for exclusion						
**n=103**			**Weak evidence**	**Focus not caregiver**	**Focus not end of life**	**Focus symptom mgmt.**	**Psycho-therapeutic intervention**	**Focus service evaluation**	**Other focus**
Addington-Hall et al 1992									
Allen et al 2008									
Anderson et al 2000									
Arnaert 2010									
Badger et al 2007									
Badger et al 2010									
Barrett et al 2009									
Baucom 2009									
Bakitas et al 2009									
Blanchard et al 1996									
Budin et al 2008									
Bull et al 2000									
Bultz et al 2000									
Campbell et al 2007									
Cameron et al 2004									
Canada et al 2005									
Carter 2006									
Christensen 1983									
Christakis and Iwashyna 2003									
Clark et al 2003									
Clark et al 2006									
Clayton et al 2007									
Cohen and Kuten 2006									
Curtis et al 2011									

Curtis et al 2013									
Dennis et al 1997									
Derdiarain 1989									
Dobrof et al 2006									
Donnolly et al 2000									
Duggleby et al 2007									
Gagnon et al 2002									
Giarelli 2003									
Given 2006									
Goldberg et al 1997									
Goldberg and Wool 1985									
Grande et al 2004									
Grant 1999									
Haley 2008									
Harding et al 2004									
Heinrich and Schag 1985									
Hendrix et al 2009									
Hoff and Haaga 2005									
Holmquist et al 2000									
Hudson et al 2005									
Hudson et al 2008									
Hudson et al 2009									
Jepson 1999									
Kane 1984									
Kayser et al 2010									
Keefe et al									
Kirk and Collins 2006									
Kissane et al 2006									
Kotila et al 1998									
Kozachik et al 2001									
Kuijer 2004									
Kurtz et al 2005									

Kusajima 2009									
Kwak et al 2007									
Lincoln et al 2003									
Lin et al 2006									
McCorkle 1998									
McCorkle 2007									
McDonald et al 2006									
McKinney et al 2002									
McLean et al 2008									
McLean et al 2011									
McLaughlin et al 2007									
McMillan et al 2006									
McMillan and Small 2007									
Magill 2009									
Mahler and Kulik 2002									
Manne 2004									
Mann and Badr 2008									
Mant et al 1998									
Meyers et al 2011									
Milberg et al 2005									
Mokuau 2008									
Mohr et al 2003									
Moser amd Dracup 2000									
Nezu et al 2013									
Northouse et al 2005									
Northouse et al 2007									
Northouse et al 2012									
Parssons and Anderson 2009									
Pelayo-Alvarez et al 2013									
Porter et al 2009									
Rodgers et al 1999									

Rudd et al 1997									
Ryan et al 2008									
Sabo et al 1986									
Schure et al 2006									
Scott et al 2004									
Shields et al 2004									
Smith et al 2004									
Scich et al 2002									
Teng et al 2003									
Thornton et al 2004									
Toselan et al 1995									
Van den Heuvel 2000 and Van den Heuvel et al 2002									
Walsh 2007									
Walsh and Smidt 2003									
Wells et al 2003									
Witkowski and Carlsson 2014									
**Totals n=103 studies**	**7**	**96**	**8**	**9**	**40**	**4**	**14**	**15**	**7**

4. Summary of papers included and excluded from each systematic review

Northouse et al (2010)

Study	Description	Included	Excluded
Badger et al 2007	Emphasis on improving communication between partners<br>Telephone-delivered psychosocial interventions and effect on depression and anxiety in women with breast cancer and their partners	No	Not end of life care
Baucom 2009	Couple based intervention for women with breast cancer	No	Psycho-therapeutic approach<br>Cognitive behavioural approach
Blanchard et al 1996	Problem solving intervention with spouses of cancer patients	No	Not end of life care
Budin et al 2008	Intervention focused on breast cancer, education, counselling and adjustment	No	Not end of life care
Bultz et al 2000	Brief psychoeducational intervention support group for partners of early stage breast cancer	No	Not end of life care
Campbell et al 2007	Focus on how African-American men cope with prostate cancer. Pilot study	No	Not end of life care
Carter (2006)	Focus on sleep problems of family caregivers	No	Not end of life care
Christensen 1983	Breast cancer counselling following mastectomy	No	Not end of life care
Derdiarain 1989	Effects of information on recently diagnosed cancer patients' and spouses' satisfaction with care	No	Not end of life care
Giarelli 2003	Caring for a spouse after prostate cancer, preparedness of wives	No	Not end of life care
Given 2006	Impact of symptom management assistance on caregiver reaction	No	Psycho-therapeutic approach<br>Cognitive behavioural approach
Goldberg and Wool 1985	Psychotherapy for the spouses of lung cancer patients	No	Psycho-therapeutic approach<br>Grief therapy

Heinrich and Schag 1985	Stress and activity management group treatment for cancer patients and spouses	No	Psycho-therapeutic approach<br>Cognitive behavioural approach
Hudson 2005	Psycho-educational intervention for family caregivers receiving palliative care	Yes	
Jepson 1999	Effects of home care on caregivers' psychosocial status	No	Not end of life care
Keefe et al	Partner-guided pain management at the end of life	No	Focused on pain management
Kissane et al 2006	Family-focused grief therapy on caregivers' bereavement outcomes	No	Psycho-therapeutic approach<br>Grief therapy
Kozachik 2001	Improving depressive symptoms among caregivers of patients with cancer	No	Not end of life care
Kuijer 2004	Brief intervention for patients with cancer and their partners	No	Psycho-therapeutic approach<br>Cognitive behavioural approach
Kurtz 2005	Patient/caregiver symptom control intervention and effects on depression in caregivers	No	Psycho-therapeutic approach<br>Cognitive behavioural approach
Manne 2004	Psychoeducational group intervention for wives of men with prostate cancer	Yes	
McCorkle 1998	Effects of home nursing care for patients during terminal illness on bereavement	No	Focused on impact of service
McCorkle 2007	Effects of advanced nursing on patient and spouse depressive symptoms, sexual function and marital interaction after radical prostatectomy	No	Not end of life care
McMillan et al 2006	Coping skills intervention for family members of hospice patients	Yes	
Mokuau 2008	Development of a family intervention for native Hawaiian women with cancer	No	Not end of life care
Northouse et al 2005	To evaluate family intervention on quality of life with women with breast cancer and family caregivers	Yes	
Northouse et al 2007	Family intervention for prostate cancer patients and spouses	Yes	

Scott et al 2004	Couple-coping intervention on adjustment in early stage breast or gynaecological cancer	No	Not end of life care
Walsh 2007	Reducing emotional distress in people caring for those receiving specialist palliative care	Yes	

Regan et al (2012)

Study	Description	Included	Excluded
Badger et al 2007	Emphasis on improving communication between partners<br>Telephone-delivered psychosocial interventions and effect on depression and anxiety of women with breast cancer and their partners	No	Not end of life care
Badger et al 2010	To test effectiveness of two telephone-delivered psychosocial interventions on QOL of men with prostate cancer and their intimate partners	No	Not end of life care
McCorkle et al 2007	To examine effects of intervention on patient and spouse depressive symptoms sexual function and marital interaction	No	Not end of life care
McLean et al 2011	To examine effect of emotionally focused therapy in advanced cancer (metastatic disease) on marital functioning and psychosocial outcomes in distressed couples	No	Psycho-therapeutic approach<br>Delivered by psychologist
Nezu et al 2003	To examine the efficacy of problem-solving therapy on psychological distress	No	Not end of life care
Kuijer et al 2004	To examine brief counselling intervention to couples – focused on social support and help both partners restoring perceptions of equity	No	Not end of life care
Porter et al 2009	To test efficacy of partner-assisted emotional disclosure intervention	No	Not end of life care
Thornton et al 2004	To test efficacy of a single-session psychological intervention integrated within pre-surgical treatment for prostate cancer	No	Not end of life care
Donnelly et al 2000	Explored feasibility of interpersonal psychotherapy by telephone to reduce distress	No	Not end of life care<br>Delivered by psychologist
Manne and Badr 2008	Intimacy-enhancing intervention for breast cancer patients and their partners	No	Not end of life care

McLean et al 2008	To evaluate effectiveness of couples' intervention in improving marital function using emotionally focused couples' therapy.	No	Psychotherapeutic approach<br>Delivered by psychologists
Mohr et al 2003	Couples therapy at the end of life, prognosis less than 18months	No	Psycho-therapeutic approach<br>Delivered by psychologists and social workers
Shields et al 2004	Focus on coping with breast cancer survivors and spouses	No	Not end of life care
Scott et al 2004	To evaluate an intervention focused on coping and adjustment to early stage breast cancer	No	Not end of life care
Northouse et al 2005	To evaluate family intervention on quality of life with women with breast cancer and family caregivers	Yes	
Northouse et al 2007	RCT family intervention for prostate cancer patients and spouses	Yes	
Kayser et al 2010	To determine the effectiveness of couple-based intervention on QOL of early stage breast cancer patients and partners	No	Not end of life care
Northouse et al 2012	To test dose of intervention needed for benefit using FOCUS intervention. Intervention delivered by master prepared nurses during three-month intervals. Usually FOCUS consists of three contacts (2 60 min home visits and a 30 min telephone call. Brief programme condensed in 3.5 hours. Extensive programme was 7 hours both 10 weeks in duration. Training of nurses 40 hours in length<br>N=848 dyads completed baseline; n-343 time 2 assessments<br>No difference between brief and extended interventions – enhanced coping in both groups	No	Methodological focus on dose effectiveness
Campbell et al 2007	Focus on how African-American men cope with prostate cancer. Pilot study	No	Not end of life care

Kozachick et al 2001	To test impact of 16-week supportive nursing intervention on caregivers of patients with newly diagnosed cancer	No	Not end of life care
Kurtz et al 2005	To test nursing intervention aimed at teaching cancer patients and their caregivers about symptom management in people with newly diagnosed solid tumours	No	Not end of life care
McCorkle et al 2007	To assess effects of advanced nursing practice on patient and spouse depression, sexual function, marital interaction following radical prostatectomy	No	Not end of life care
Budin et al 2008	RCT to test psychoeducation and telephone counselling on adjustment of women with breast cancer and their partners	No	Not end of life care

Waldron et al (2013)

Study	Description	Included	Excluded
Campbell et al 2007	Focus on how African-American men cope with prostate cancer. Pilot study	No	Not end of life care
Carter (2006)	Focus on reported sleep problems of family caregivers	No	Not end of life care
McMillan et al 2006	Coping skills intervention family members of hospice patients	Yes	
Northouse et al 2005	To evaluate family intervention on quality of life with women with breast cancer and family caregivers	Yes	
Northouse et al 2007	RCT family intervention for prostate cancer patients and spouses	Yes	
Walsh et al 2007	Reduction emotional distress in carers of those receiving palliative care	Yes	

Candy et al (2011)

Study	Description	Included	Excluded
Addington-Hall 1992	Controlled trial of co-ordinating care for terminally ill cancer patients	No	Not caregiver focused
Allen 2008	Controlled trial of legacy activities such as scrapbooking and audio stories	No	Psycho-therapeutic approach<br>Life review and grief work
Carter (2006)	Focus on reported sleep problems of family caregivers	No	Not end of life care
Hudson 2005		Yes	
Kane 1984	Controlled trial of hospice care	No	Focused on service evaluation
Keefe 2005		No	Focused on pain control
Kissane 2006		No	Psycho-therapeutic approach<br>Grief therapy
McMillan et al 2006	Coping skills intervention for family members of hospice patients	Yes	
Northouse et al 2005	To evaluate family intervention on quality of life with women with breast cancer and family caregivers	Yes	
Northouse et al 2007	RCT family intervention for prostate cancer patients and spouses	Yes	
Walsh et al 2007	Reduction emotional distress in carers of those receiving palliative care	Yes	

Nevis (2014)

Study	Description	Included	Excluded
Pelayo-Alvarez et al 2013	Clinical effectiveness of an on-line training programme for palliative care physicians	No	Other focus - training physicians
Curtis et al 2013	Effect of training skills training for residents and nurse practitioners	No	Other focus - communication skills training
Curtis et al 2011	Effect of a quality-improvement intervention on end of life care in intensive care	No	Focused on service development
Meyers et al 2011	Effects of COPE on QOL for patients with advanced cancer and their family caregivers	No	Other focus - participants in clinical trials
Bakitas et al 2009	Effects of ENABLE II on clinical outcomes in patients with advanced cancer	No	Not caregiver focused
McMillan et al 2006	Coping skills intervention for family members of hospice patients	Yes	

Harding et al (2012)

Study	Description	Included	Excluded
Cameron et al 2004	To enhance problem solving abilities of carers and confidence in their role	No	Graded as weak evidence by reviewers
Hudson et al 2009	Evaluation of benefits of MDT guidelines in family meetings. Meetings were led by nurses, trained to use the guidelines. Family concerns assessed pre and post meeting. The family meetings significantly reduced carers worry, and their concerns interfered less with their life	No	Graded as weak evidence by reviewers<br><br>Learning point: Helpful study re focus on family member concerns and influence of a nurse-led meeting on these concerns
Hudson et al 2005	Psycho-educational intervention for family caregivers receiving palliative care	Yes	
Milberg et al 2005	To enhance support and knowledge – individualised intervention provided by specialist nurses	No	Graded as weak evidence by reviewers
Walsh and Smidt 2003	Telecare intervention for carers of hospice patients - pilot study	No	Graded as weak evidence by reviewers
Walsh et al 2007	Focus on reducing emotional distress in carers of those receiving palliative care	Yes	
Carter (2006)	Focus on reported sleep problems of family caregivers	No	Not end of life
Ryan et al 2008	Non-clinical community based social support for carers of people with lung cancer	No	Graded as weak evidence by reviewers

Magill 2009	Home based palliative care music therapy, effect of providing before bereavement on post-bereavement outcomes	No	Other focus - music therapy
Northouse et al 2007	RCT family intervention for prostate cancer patients and spouses	Yes	
Allen et al 2008	Family-based intervention to reduce caregiving stress	No	Psycho-therapeutic approach<br>Life review and grief work
Northouse et al 2005	To evaluate family intervention on quality of life with women with breast cancer and family caregivers	Yes	
Christakis and Iwashyna 2003	To evaluate spouse mortality after bereavement, large matched retrospective cohort study	No	Focused on service
Kirk and Collins 2006	Evaluation of hospital palliative care team in Africa	No	Focused on service
Kusajima 2009	Evaluation of the transition to specialist home palliative care – prospective pre and post evaluation	No	Focused on service
Grande et al 2004	RCT hospice at home service	No	Focused on service
McLaughlin et al 2007	Evaluation of a hospice at home service	No	Focused on service
Clayton et al 2007	RCT Evaluation of question prompt training for patients and caregivers	No	Other focus - training patients and caregivers to optimise consultations
Hendrix et al 2009	Evaluation of training for patients and caregivers in symptom management	No	Focused on symptom management
Dobrof et al 2006	Retrospective evaluation of social work provision	No	Focused on service
Barrett et al 2009	Evaluation of an at-home respite service	No	Focused on service
McMillan et al 2006	Coping skills intervention family members of hospice patients	Yes	

Parsons and Anderson 2009	Ethnographic interview study of afternoon tea sessions for patients and families on a palliative care unit	No	Focused on service
Cohen and Kuten 2006	Comparison between group support, intervention group used Cognitive behavioural therapy	No	Psycho-therapeutic approach<br>Cognitive behavioural approach
Witkowski and Carlsson 2014	Phenomenological study of group support session	No	Focused on service
Kwak et al 2007	Evaluation of a caregiver training programme	No	Other focus – caregiver training
Arnaert 2010	Evaluation of a weekend retreat for caregivers	No	Focused on service
Hudson et al 2008	Pre- and post-evaluation of a 3-week group psychoeducational intervention for preparation of caregivers	No	Other focus – caregiver training
Hudson et al 2009	Evaluation of benefits of MDT guidelines in family meetings. Meetings were led by nurses, trained to use the guidelines. Family concerns assessed pre and post meeting. The family meetings significantly reduced carers worry, and their concerns interfered less with their life	No	Graded as weak evidence by reviewers<br><br>Learning point: Helpful study re focus on family member concerns and influence of a nurse-led meeting on these concerns
Clark et al 2006	Evaluation of an 8 session structured MDT intervention for cancer patients designed to improve carer's burden and QOL	No	Not caregiver focused
McDonald et al 2006	Evaluation of a 12-week hospice yoga programme for patients	No	Not caregiver focused

Chambers et al 2011 – studies included in review identified as addressing caregiver needs

Study	Description	Included	Excluded
Campbell et al 2007	Focus on how African-American men cope with prostate cancer. Pilot study	No	Not end of life care
Canada et al 2005	Pilot intervention to enhance couple sexual rehabilitation following treatment for prostate cancer	No	Other focus - sexual rehabilitation
Manne 2004	Psychoeducational group intervention for wives of men with prostate cancer	Yes	
McCorkle 2007	Effects of advanced nursing on patient and spouse depressive symptoms, sexual function and marital interaction after radical prostatectomy	No	Not end of life care
Northouse et al 2007	RCT family intervention for prostate cancer patients and spouses	Yes	

Caress et al (2009) studies identified as focused on symptom management excluded (n=10)

Study	Description	Included	Excluded
McCorkle et al 2007	To examine effects of specialised home care intervention on survival of older post-surgical patients	No	Not end of life care
Cameron et al 2004	To enhance problem solving abilities of carers and confidence in their role	No	Graded as weak evidence by Harding et al 2012 and Ussher et al 2009
Kozachick et al 2001	To test impact of 16-week supportive nursing intervention on caregivers of patients with newly diagnosed cancer	No	Not end of life care
McMillan and Small 2007<br>McMillan et al 2006	Coping skills intervention family members of hospice patients	Yes	
Nezu et al 2003	To examine the efficacy of problem-solving therapy on psychological distress	No	Not end of life care
Toselan et al 1995	**Evaluation of a problem-solving intervention provided over 6 x 1hour sessions RCT, provided by an oncology social worker.**	No	Not end of life care<br><br>Learning points: steps in the Problem-solving model included:<br>Identifying the problem<br>Generating alternative solutions<br>Examining the benefits and drawbacks of each solution<br>Discussing and rehearsing an action plan<br>Evaluating the plan
Harding et al 2004	Evaluation of teaching programme for carers of people receiving home-based palliative care	No	Other focus - teaching programme

Hudson 2005	Psycho-educational intervention for family caregivers receiving palliative care	Yes	
Hudson et al 2008	Evaluation of a psycho-educational group programme for family caregivers in home-based palliative care	No	Graded as weak evidence by reviewers
Walsh 2007	Reducing emotional distress in people caring receiving specialist palliative care	Yes	

Ussher et al 2009

Study	Description	Included	Excluded
Christensen 1983	Breast cancer counselling following mastectomy	No	Not end of life care
Kissane et al 2006	Family-focused grief therapy on caregivers bereavement outcomes	No	Psychotherapeutic approach<br>Grief therapy
Blanchard et al 1996	Problem solving intervention with spouses of cancer patients	No	Not end of life care
Bultz et al 2000	Brief psychoeducational intervention support group for partners of early stage breast cancer	No	Not end of life care
Walsh et al 2007	Reduction emotional distress in carers of those receiving palliative care	Yes	
Toselan et al 1995	Evaluation of a problem-solving intervention provided over 6 x 1hour sessions RCT, provided by an oncology social worker.<br>Steps in the Problem-solving model included:<br>Identifying the problem<br>Generating alternative solutions<br>Examining the benefits and drawbacks of each solution<br>Discussing and rehearsing an action plan<br>Evaluating the plan	No	Not end of life care
Northouse et al 2005	To evaluate family intervention on quality of life with women with breast cancer and family caregivers	Yes	

Scott et al 2004	To evaluate an intervention focused on coping and adjustment to early stage breast cancer	No	Not end of life care
Hudson 2005	Psycho-educational intervention for family caregivers receiving palliative care	Yes	
Kozachick et al 2001	To test impact of 16-week supportive nursing intervention on caregivers of patients with newly diagnosed cancer	No	Not end of life care
Goldberg and Wool 1985	Psychotherapy for the spouses of lung cancer patients	No	Psychotherapeutic approach<br>Grief therapy
Kuijer et al 2004	To examine brief counselling intervention to couples – focused on social support and help both partners restoring perceptions of equity	No	Not end of life care
Kayser 2005	Couple psychosocial intervention over 9 sessions RCT for women with breast cancer currently receiving treatment and their partners	No	Not end of life care
McCorkle 1998	The effects of home nursing care for patients during terminal illness on bereavement	No	Focused on service
Heinrich and Schag 1985	Stress and activity management group treatment for cancer patients and spouses	No	Psychotherapeutic approach<br>Cognitive behavioural approach
Gagnon et al 2002	Psychoeducation re. delirium	No	Focused on symptom management
Sabo et al 1986	Evaluation of facilitated support group for men following partner treatment with mastectomy for breast cancer	No	Not end of life care

Glasdam et al 2010

Study	Description	Included	Excluded
Anderson et al 2000	Comparison of home or hospital rehabilitation therapy following stroke	No	Focused on service
Clark et al 2003	RCT evaluating whether education and counselling following stroke influenced physical functioning and social outcomes	No	Not end of life care
Dennis et al 1997	Evaluation of a stroke family worker	No	Focused on service
Goldberg et al 1997	Evaluation of home-based case-managed care for stroke survivors	No	Not end of life care
Grant 1999	Pilot study comparison of effectiveness of home and telephone social problem-solving partnerships on family carer outcomes and influence of stroke survivor characteristics	No	Not end of life care
Grant et al 2002	Evaluation of a telephone support intervention with caregivers of stroke survivors	No	Not end of life care
Van den Heuvel 2000 and Van den Heuvel et al 2002	Evaluation of an exercise programme vs education home visits vs no intervention	No	Not end of life care
Holmquist et al 2000	Comparison of home rehabilitation group vs hospital rehabilitation	No	Not end of life care
Kotila et al 1998	Incidence of depression after stroke	No	Not end of life care
Lincoln et al 2003	Evaluation of a stroke family support organizer	No	Focused on service

Mant et al 1998	RCT of impact of information pack on family members of people recovering from stroke	No	Not end of life care
Mant et al 2005	One year follow up of RCT above	No	Not end of life care
McKinney et al 2002	Evaluation of cognitive assessment in stroke	No	Not end of life care
Rodgers et al 1999	Randomised trial of a stroke education programme	No	Not end of life care
Rudd et al 1997	RCT evaluation of early discharge for people following stroke	No	Not focused on caregivers
Schure et al 2006	Evaluation of group support vs home visiting programme following method outlined by Van den Heuvel	No	Not end of life care
Smith et al 2004	Evaluation of a stroke recovery programme	No	Not focused on end of life care
Sulch et al 2002	Impact of integrated care pathway on quality of life for stroke	No	Not focused on caregivers
Teng et al 2003	Evaluation of home education group vs standard discharge and follow up re costs for caregivers of early discharge	No	Not focused on end of life care
Bultz et al 2000	Brief psychoeducational intervention support group for partners of early stage breast cancer	No	Not end of life care specific
Cohen and Kuten 2006	Comparison between group support, intervention group used CBT	No	Psychotherapeutic approach<br>Cognitive behavioural approach
Given 2006<br>Kurtz et al 2005	Impact of symptom management assistance on caregiver reaction RCT	No	Cognitive behavioural approach
Hoff and Haaga 2005	Effect of an education programme on radiation oncology patients and families	No	Not end of life care
Kozachick et al 2001	To test impact of 16-week supportive nursing intervention on	No	Not end of life care

	caregivers of patients with newly diagnosed cancer		
Kuijer et al 2004	To examine brief counselling intervention to couples – focused on social support to help both partners restore perceptions of equity	No	Not end of life care
Lin et al 2006	Evaluation of long-term effectiveness of family and patient pain education programme	No	Not end of life care
Northouse et al 2005	To evaluate family intervention on quality of life with women with breast cancer and family caregivers	Yes	
Scott et al 2004	To evaluate an intervention focused on coping and adjustment to early stage breast cancer	No	Not end of life care
Wells et al 2003	Education to improve pain management for patients and caregivers	No	Not end of life care
Bull et al 2000	To examine differences in outcomes for elders and caregivers who participated in a professional - partnership model of discharge for patients with heart failure	No	Not end of life care
Mahler and Kulik 2002	Evaluation of videotaped information on spousal distress following surgery	No	Not end of life care
Moser and Dracup 2000	Evaluation of group education about resuscitation	No	Other focus - training needs

Hudson et al 2010

Study	Description	Included	Excluded
McMillan et al 2006	Coping skills intervention family members of hospice patients	Yes	
Hudson 2005	Psycho-educational intervention for family caregivers receiving palliative care	Yes	
Walsh et al 2007	Reduction emotional distress in carers of those receiving palliative care	Yes	
Keefe 2005		No	Focused on pain control
Haley 2008		No	Not end of life care
Harding et al 2004	Evaluation of teaching programme for carers of people receiving home-based palliative care	No	Graded as weak evidence by reviewers
Hudson et al 2008	Pre- and post-evaluation of a 3-week group psychoeducational intervention for preparation of caregivers	No	Graded as weak evidence by reviewers
Hudson et al 2009	Evaluation of benefits of MDT guidelines in family meetings. Meetings were led by nurses, trained to use the guidelines. Family concerns assessed pre and post meeting. The family meetings significantly reduced carers worry, and their concerns interfered less with their life	No	Graded as weak evidence by reviewers<br><br>Learning point: helpful study re focus on family member concerns and influence of a nurse-led meeting on these concerns
Carter (2006)	Focus on reported sleep problems of family caregivers	No	Not end of life care<br>Graded as weak evidence by reviewers
Kwak et al 2007	Evaluation of a programme focusing on closure during end of life care	No	Graded as weak evidence by reviewers

Duggleby et al 2007	Development of a living hope programme for caregivers of family members with advanced cancer	No	Graded as weak evidence by reviewers
Walsh and Smidt 2003	Telecare intervention for carers of hospice patients - pilot study	No	Graded as weak evidence by reviewers and by Harding et al 2012
Milberg et al 2005	To enhance support and knowledge – individualised intervention provided by specialist nurses	No	Graded as weak evidence by reviewers and by Harding et al 2012

6. Comparison of COPE and FOCUS from analysis of intervention manuals using checklist for summarising psychological interventions (Hodges et al 2011)

	COPE (McMillan and colleagues)	FOCUS (Northouse and colleagues)
**Context**<br><br>**Indications**: Conditions under which the intervention was used (e.g. presence of diagnosis or symptoms)<br><br>**Population**: Inclusion and exclusion criteria for people receiving the intervention<br><br>**Location**: The geographical location and the type of service in which the intervention was tested.	Symptoms related to advanced cancer<br><br>Family caregivers of people with advanced cancer (hospice)<br>Inclusion (in trial) Adults, cancer, family caregiver, literate, cognitively intact<br><br>South Florida Hospice Care	Coping with prostate cancer<br>Couples as an emotional system<br><br>Men and family members with prostate cancer at all stages of illness<br>Later studies (than that reported in manual) have used FOCUS with people with breast cancer and advanced cancer<br>Age above 30yrs, partner age above 21<br><br>Home, US
**Domain I Intervention content**<br>**Components**<br><br>**Techniques**<br>**Treatment Materials**<br><br>**Tailoring to individual patients**	Components: Creativity; Optimism; Planning and Expert Information<br><br>Treatment materials: caregiving guide (how to manage 23 common symptoms) available - http://www.cancer.org/treatment/treatmentsandsideeffects/physicalsideeffects/dealingwithsymptomsathome/caring-for-the-patient-with-cancer-at-home-intro<br>Tailoring by initial identification of issues of concern	Components: family involvement, optimistic attitude, coping effectiveness, uncertainty reduction, symptom management<br><br>Treatment materials: various related to information and coping and symptom management – pt booklets, drug information sheets, symptom management sheets, relaxation audio tape<br><br>Tailoring to issues of concern but intent to cover all issues identified in research protocol
**Domain 2 Proposed mechanisms**		

**Mechanisms of action**	Enhanced problem solving<br>Use of expert practitioners to help solve problems	Enhanced problem solving<br>Enhanced communication between patient and partner
**Domain 3 Target outcomes**<br>**Primary outcome**<br>**Secondary outcome**	 Primary: Improved caregiver QOL, reduced caregiver distress from patient symptoms (MSAS-CG), coping (brief COPE)<br>Secondary: decreased patient symptom distress	 Primary: reduced uncertainty for partner; enhanced communication between pt and partner<br>Secondary: increased QoL for partner; increased appraisal of ability as caregiver; increased self-efficacy; reduced symptom distress patient
**Domain 4 Method of delivery**<br>**Delivery setting and method**<br><br>**Timing of treatment**<br>**Therapist**<br>**Training**	 Delivery face-to-face over 3 sessions in patient's home over 9 days<br>Session 1: 45mins; session 2: 30mins; session 3: 30mins<br>Hospice RN<br>8hrs training inclusive of instruction and role play plus 3 pilot interventions, audiotaped and assessed for fidelity	 3 structured 90 min visits face-to-face with telephone booster between visits of 30mins<br><br><br>40 hours training for master level RN's using training video, talks and shadowing experienced nurses
Comments	Prompts clear for intervention; topic discussion clear<br>Home care assistant looking after patient whilst nurse delivering intervention	
Reflections	Long interventions in terms of time taken to deliver – several successive consultations<br>Later studies with FOCUS compared 30min interventions with standard length and found no difference in outcomes<br>Later study with COPE with people with heart failure did not replicate outcomes of previous studies – thought carer more experienced in issues related to illness so intervention might have been more helpful earlier in illness<br>Resource intensive<br>Both interventions focused on coping whilst at home<br>Focus tested in advanced cancer, similar results – also investigated risk for distress screening tool (omega clinical screening tool) but found all caregivers were at risk of distress	

Additional source:

McMillan http://www.rosalynncarter.org/UserFiles/File/2009_asa_ncoa_conference/mcmillan.pdf

COPE:

C: Creativity – viewing problems from different perspectives to develop new strategies for solving caregiving problems

O: Optimism – having a positive but realistic attitude toward solving the problem – includes communicating realistic optimism to patient, showing optimism and hope

P: Planning – setting out reasonable goals and the steps needed to reach goals

E: Expert Information – what caregivers need to know about a problem, when to get help and what they can do to help themselves manage the problem

7. Summary of analysis of papers included in evidence review

Study	Guiding framework	Inputs (resources)	Activities	Outputs<br>N=<br>Sample characteristics	Intermediate outcomes	Outcomes
Hudson et al 2005	Lazarus and Folkman's transactional model of stress and coping	Nurse<br><br>Caregiver guidebook and audiotape	2 home visits and one follow phone call<br><br>Provide opportunity to access information and provide basis for skill acquisition<br><br>Reinforcement role of p/c services and other services and providing strategies for involving family and friends<br><br>Helping caregiver find meaning in situation, normalising emotional reactions, encouraging them to see positive aspects of experience, offering spiritual guidance<br><br>Encouraging carer to take care of themselves<br><br>Providing advice on carer's rights	N=106; 54 received intervention; 52 received standard care<br><br>Mean age 60.78<br>67% women carers<br>Advanced cancer<br>Australian	Preparedness for caregiving<br><br>Perceived competence as caregiver<br><br>Perceived potential benefits of caring<br>Mastery (perceived control over events)<br><br>Psychological distress (HAD scale)	Increased satisfaction/Reward of caregiving
Manne et al 2004	Lazarus Stress and Coping theory<br>Horowitz Cognitive and social processes	Clinical experts related to session focus	Group intervention consisting of 6 x 1 hour sessions, with a different topic for each session, plus homework after session 3 and 4 – sessions consisted of didactic and group contributions<br><br>Finding benefit and meaning in experience; how to recognise support	N=128 women married to men with prostate cancer approached, n=68 agreed to participate; final sample size n=60 – (n=7 dropped out) randomized into 2 groups, n=29	Coping skills<br>Open communication and social support<br><br>Psychological distress (Mental Health Inventory and Impact Events Scale)	Personal strength, spiritual growth and appreciation for life

	theory of adaptation		needs and get these met; how to recognise support that is effective and ineffective; skills training in effective communication	intervention, 31 control group participants<br><br>Mean age 59.63 years	Marital communication<br><br>Post traumatic Growth	
McMillan et al 2006<br><br>And McMillan and Small, 2007	Implicit stress and coping therapy	Nurse	Usual hospice care + Problem-focused skills teaching with 4 components: Creativity; optimism; planning; lay info provision<br><br>COPE	N=354 eligible for participation; 329 randomized (3 armed RCT)<br>Intervention group: n=111; enhanced usual care: n=109; control group usual care: n=109<br>Large number of carers dropped out before programme completed<br>63-71% pt decline in health and carer feeling overwhelmed<br><br>Mean age approx. 60<br>Most female<br>Cancer, hospice programme<br>USA	Carer mastery<br>Burden<br>Burden of cancer symptoms	QOL<br><br>Carer coping
Northouse 2005	Stress appraisal model from Lazarus and colleagues	Nurse	3 monthly visits around 1.5 hours long<br>Follow up telephone calls total 5 contacts<br><br>FOCUS ?intervention – information and support<br><br>5 components: promoting cohesive family; encouraging optimistic outlook; coping effectiveness; provision	182 families of which 96 received intervention<br>134/182 follow up assessments completed; no difference in drop out between intervention and control group<br><br>Mean age 52<br>62% husbands	Coping effectiveness<br>Hopelessness reduction<br>Reduction uncertainty<br>Appraisal of illness	QOL

			information to reduce uncertainty; assist carer to manage symptoms	Breast cancer		
Northouse 2007	As above	Nurse	3 x 90-minute home visits + 2 phone calls 2 weeks apart<br><br>FOCUS intervention	263 couples<br>84% Caucasian 14% African Americans<br><br>Prostate cancer<br>Mean age 59<br><br>218/263 couples completed all three sessions, attrition due to death of pt (15), business of carer (6)	Functional Assessment of Cancer (FACT-P)<br>Appraisal of illness<br>Appraisal of caregiving<br>Uncertainty<br>Hopelessness<br>Coping strategies<br>Symptom distress	QOL
Walsh 2007	Not explicitly stated	Trained advisors – trained nurse and trained social worker	6 weekly sessions<br>Specialist p/c cf care by trained advisors (intervention)	271<br><br>British<br>Mostly women n=215<br>Mean age 56.4<br>Cancer<br><br>54/72 completed follow up intervention group<br>69/90 in control group	Caregivers psychological distress<br>Carer strain<br>Satisfaction with care	QOL
