# Supplementary material for: Co-construction of the family-focused support conversation: a participatory learning and action research study to implement support for family members whose relatives are being discharged for end-of-life care at home or in a nursing home
Source: BMC Palliat Care. 2020 Sep 21;19:146. doi: 10.1186/s12904-020-00647-5 (PMC7507823; doi:10.1186/s12904-020-00647-5)
Supplement: Supplementary file 3 — Additional file 3: Supplementary information 3. Description of Family-Focused Support Conversation using TIDieR checklist (Hoffman et al, [65]). [file 12904_2020_647_MOESM3_ESM.docx]

**Supplementary information 3: Description of Family-Focused Support Conversation using TIDieR checklist (Hoffman et al, 2014)**

| 1 | What | Name or a phrase that describes the intervention | The Family-Focused Support Conversation (FFSC): |
| --- | --- | --- | --- |
| 2 | Why | Rationale, theory, or goal of the elements essential to the intervention | A brief intervention to support family members during end of life care transitions – between care in hospital and care at home of nursing home for a dying relative. End of life care transitions can be distressing for family members (Payne and Morby, 2013) because they signify deterioration and the impending death of their ill relative and forthcoming bereavement (Penrod et al, 2012). However, there is limited evidence about how to provide such support during care transitions (Candy et al, 2011, Thomas et al, 2017) and consequently, family members report an absence of support from hospital-based healthcare professionals (Hanratty et al, 2014). |
| 3 | Materials | Describe any physical or informational materials used in the intervention, including those provided to participants or used in intervention delivery or in training of intervention providers. Provide information on where the materials can be accessed (such as online appendix, URL) | The intervention is delivered via a conversational process between practitioner and family member. Small prompt cards were used by practitioners as an aide memoire to the process of the intervention, prior to talking to the family member.  Training was provided via facilitated 2-hour group session, outlining the intervention, the process and underpinning theoretical basis. Training is supported by a training pack. |
| 4 | Procedures | Describe each of the procedures, activities, and/or processes used in the intervention, including any enabling or support activities | The intervention comprises 3 components, which are woven into the normal process of a conversation by the practitioner asking family-focused questions (the interventional process is provided in brackets). Full details of the components, conversational prompts and interventional processes are provided in table 2 of the paper and in training pack):  Meaningfulness – the significance of the care transition for the family (impact)  Comprehensibility – the sense the family are making of the care transition (implications)  Manageability – the resources the family can draw upon and how they might organise the impact and implications of the care transition between them. |
| 5 | Who provided | For each category of intervention provider (such as psychologist, nursing assistant), describe their expertise, background, and any specific training given | The intervention is intended to be used by health care professionals working in NHS hospitals |
| 6 | How | Describe the modes of delivery (such as face to face or by some other mechanism, such as internet or telephone) of the intervention and whether it was provided individually or in a group | The intervention can be delivered face-to-face and by telephone, usually individually with a family member, or a family group. |
| 7 | Where | Describe the type(s) of location(s) where the intervention occurred, including any necessary infrastructure or relevant features | The intervention can be delivered in wherever a conversation is taking place with a family member. |
| 8 | When and how much | Describe the number of times the intervention was delivered and over what period of time including the number of sessions, their schedule, and their duration, intensity, or dose | The intervention is designed to be delivered once, either in one meeting or spread across successive consultations. |
| 9 | Tailoring | If the intervention was planned to be personalised, titrated or adapted, then describe what, why, when, and how | The intervention is personalised according to concerns and worries raised by family members, as above. |
| 10 | Modifications | If the intervention was modified during the course of the study, describe the changes (what, why, when, and how) | The intervention was modified during the study by practitioner co-researchers as described in the accompanying paper. |
| 11 | How well: planned | If intervention adherence or fidelity was assessed, describe how and by whom, and if any strategies were used to maintain or improve fidelity, describe them | Practitioners were asked to complete a reflective sheet at the end of each intervention, to record the extent to which they had incorporated the interventional components and whether the intervention had flagged up any delivery difficulties. Regular support telephone calls between co-researchers and research team members were scheduled to discuss intervention delivery and its process. Frequency and participation in these calls varied to suit the teams. |
| 12 | How well: actual | If intervention adherence or fidelity was assessed, describe the extent to which the intervention was delivered as planned | Co-researchers used the intervention in the way that was congruent with the outlined process – further details in the accompanying paper. |
